# Clinically Amyopathic Dermatomyositis With Rapid Progressive Interstitial Lung Disease Diagnosed in a Patient on Extracorporeal Membrane Oxygenation

**DOI:** 10.7759/cureus.27839

**Published:** 2022-08-10

**Authors:** Faisal Al-Husayni, Adeeb Munshi, Sultan Qanash, Talal A Shaikhain, Zeyad Alzahrani, Bader Alghamdi

**Affiliations:** 1 Internal Medicine, King Abdullah International Medical Research Center, Jeddah, SAU; 2 Internal Medicine, National Guard Hospital, King Abdulaziz Medical City, Jeddah, SAU; 3 Medicine/Infectious Diseases, King Abdullah International Medical Research Center, Jeddah, SAU; 4 Infectious Diseases, King Saud bin Abdulaziz University for Health Sciences, Jeddah, SAU; 5 Infectious Diseases, King Abdulaziz Medical City, Jeddah, SAU; 6 Internal Medicine, King Abdulaziz Medical City, Jeddah, SAU; 7 Rheumatology, King Abdulaziz Medical City, Jeddah, SAU; 8 Pulmonary, King Abdulaziz Medical City, Jeddah, SAU; 9 College of Medicine, King Saud bin Abdulaziz University for Health Sciences, Jeddah, SAU; 10 Pulmonary, King Abdullah International Medical Research Center, Jeddah, SAU

**Keywords:** anti-mda-5, rapid progressive interstitial lung disease, lung biopsy, extracorporeal membrane oxygenation, clinically amyopathic dermatomyositis

## Abstract

Clinically amyopathic dermatomyositis (CADM) is characterized by skin manifestations with minimal to no muscle involvement. It is a unique subset of dermatomyositis, which may create a diagnostic challenge due to its vague presentation. Establishing the diagnosis is crucial as CADM is highly associated with rapidly progressive interstitial lung disease (RP-ILD), and patients who suffer from thereof have an abysmal prognosis. Herein, we described a case of a 46-year-old male who presented with a history of skin rash and then started to experience shortness of breath. His respiratory symptoms were progressing swiftly and affected his daily life activities. The initial blood tests were normal, but his chest imaging revealed fibrotic nonspecific interstitial pneumonia. The patient required intubation due to a critical respiratory condition, and later, he needed extracorporeal membrane oxygenation (ECMO). While the patient was connected to an ECMO machine, a bedside open lung biopsy (BOLB) was performed, and the results were in keeping with RP-ILD and CADM. The patient was started on cyclophosphamide without a response, and his chest computed tomography showed acute respiratory distress syndrome. His hospital course was complicated with pneumonia, severe kidney dysfunction requiring dialysis, and candidemia, which resulted in the patient’s death.

## Introduction

Dermatomyositis (DM) is an idiopathic inflammatory disease featured by skin and muscle involvement [1]. The pathophysiology of DM is thought to be due to complement deposition in the blood vessels. The diagnosis of DM can be challenging, but classically, patients will exhibit skin manifestations, including Gottron’s papules and the Heliotrope eruption, in addition to evidence of muscle injury [2]. However, the absence of muscular manifestations may raise the suspicion of clinically amyopathic dermatomyositis (CADM).

CADM is a unique subtype of idiopathic inflammatory myopathies characterized by dermatological manifestations with minimal or absent myopathy [3-5]. It compromises around 20% of all DM cases with female predilection [6,7]. The clinical presentation and prognosis vary based on several autoantibodies. Anti-melanoma differentiation-associated gene 5 (anti-MDA-5) antibody has been linked to inauspicious outcomes in patients with CADM [8,9].

High mortality and morbidity rates in CADM patients are attributed to concomitant rapid progressive interstitial lung disease (RP-ILD) [3,4]. Thus, it is crucial to establish the type of ILD and identify the different biomarkers, especially in patients presenting with cutaneous involvement. Many studies have suggested that anti-MDA-5 antibodies are associated with CADM-related RP-ILD [10]; nonetheless, a patient may still procure anti-MDA-5 antibody and RP-ILD without demonstrating skin or muscle involvement [11,12].

Herein, we present a case of a young gentleman who presented with skin rash and severe respiratory failure. The patient was diagnosed with CADM-related RP-ILD by obtaining a bedside open lung biopsy (BOLB) while being on extracorporeal membrane oxygenation (ECMO).

## Case presentation

**Figure 1:**
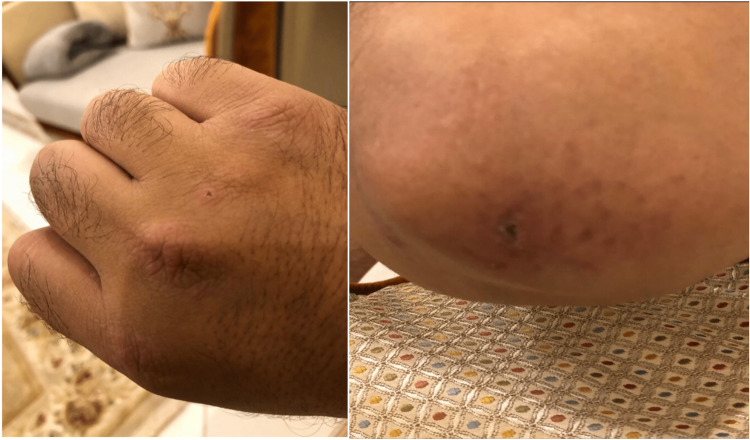
Erythematosus flat-topped papules coalesce to form plaques with some excoriation affecting the elbow and dorsal part of the hand

In August of 2018, a previously healthy 46-year-old male presented with a two-week history of severe dyspnea. Four months before his presentation, he started to have mild itching in the lower limbs and abdomen. The itching used to occur yearly at the beginning of the summer season, which responded well to anti-histamine. Three months before presenting to the hospital, the patient experienced mild cough and shortness of breath resembling New York Heart Association (NYHA) class II, which he attributed to his smoking habits as he is an occasional vape user; thus, he did not seek medical attention. Two months before his presentation, he started to have intermittent subjective fever, night sweating, ankle and wrist joint pain, decreased appetite, and skin rash over the hands (Figure 1).

**Figure 2:**
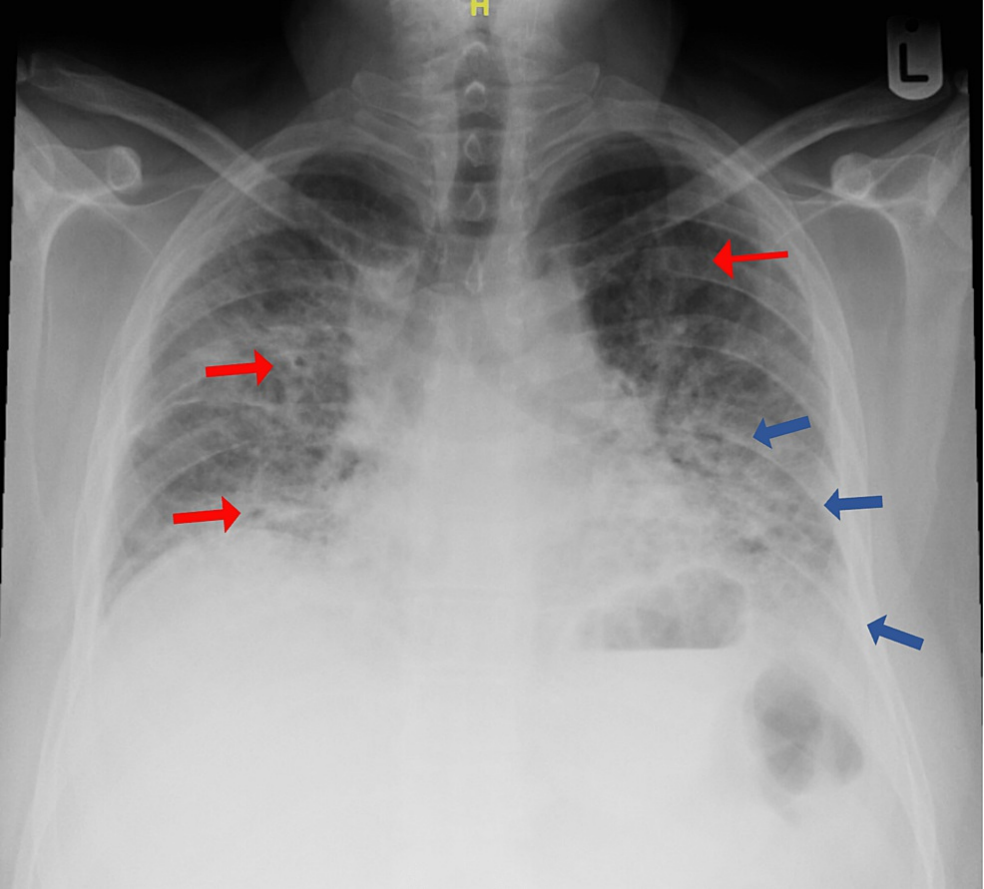
Patient’s chest x-ray showing bilateral cystic changes (red arrows) with patchy airspace opacities (blue arrows) mostly appreciated in the left lower lobe and left retrocardiac area

Two weeks before his presentation, his shortness of breath worsened (NYHA class III), and as it persisted, he sought medical advice. Initially, the patient had normal vital signs, including normal oxygen saturation on ambient air. The lung examination revealed decreased breath sounds bilaterally with coarse crepitations. The rash was present on the extensor areas of both hands, elbows, and soles. The rest of the examination was unremarkable. Basic blood work, such as complete blood count (CBC), renal function test (RFT), liver function test (LFT), coagulation profile, bone panel creatinine kinase (CK), and cardiac markers, was normal. Chest x-ray (CXR) demonstrated bilateral cystic changes with multiple patchy areas of airspace opacities most appreciated in the left lower lobe and left retrocardiac area (Figure 2).

A small left pleural effusion was noted with normal cardiac size. The patient was started on moxifloxacin and methylprednisolone 60 mg once daily. Microbiological cultures, viral panel, and immunological workup were tested and resulted negative (Table 1).

**Table 1:** Patient’s immunological panel MPO: Myeloperoxidase antibodies; PR3: Proteinase 3.

Lab	Results	Reference range
Antinuclear antibody	Positive	Negative
Anti-double stranded DNA	66.8 IU/mL	<68.6 is negative; 68.6-229 is moderately positive; >229 is strongly positive
Rheumatoid factor	<10.70 IU/M	<15 IU/ML
Ribonucleoprotein antibody	4.30 U	<20 is negative; 20-39 is weakly positive; 40-80 is moderately positive; >80 is strongly positive
Anti-Smith antibody	4.08 U	<20 is negative; 20-39 is weakly positive; 40-80 is moderately positive; >80 is strongly positive
Antineutrophil cytoplasmic PR3 antibody (C-ANCA)	8.28 U	<20 is negative; 21-30 is weakly positive; >30 is moderate to strongly positive
Antineutrophil cytoplasmic MPO antibody (P-ANCA)	2.60 U	<20 is negative; 21-30 is weakly positive; >30 is moderate to strongly positive
Anti-RO	27.15 U	<20 is negative; 20-39 is weakly positive; 40-80 is moderately positive; >80 is strongly positive
Anti-LA	3.21 U	<20 is negative; 20-39 is weakly positive; 40-80 is moderately positive; >80 is strongly positive
Anti-JO	4.35 U	<20 is negative; 20-39 is weakly positive; 40-80 is moderately positive; >80 is strongly positive
Anti-SCL70 antibodies	5.35 U	<20 is negative 20-39 is weakly positive 40-80 is moderately positive >80 is strongly positive
C3 complement	1.55 g/L	0.9-1.9 g/L
C4 complement	0.37 f/L	0.1-0.4 g/L

**Figure 3:**
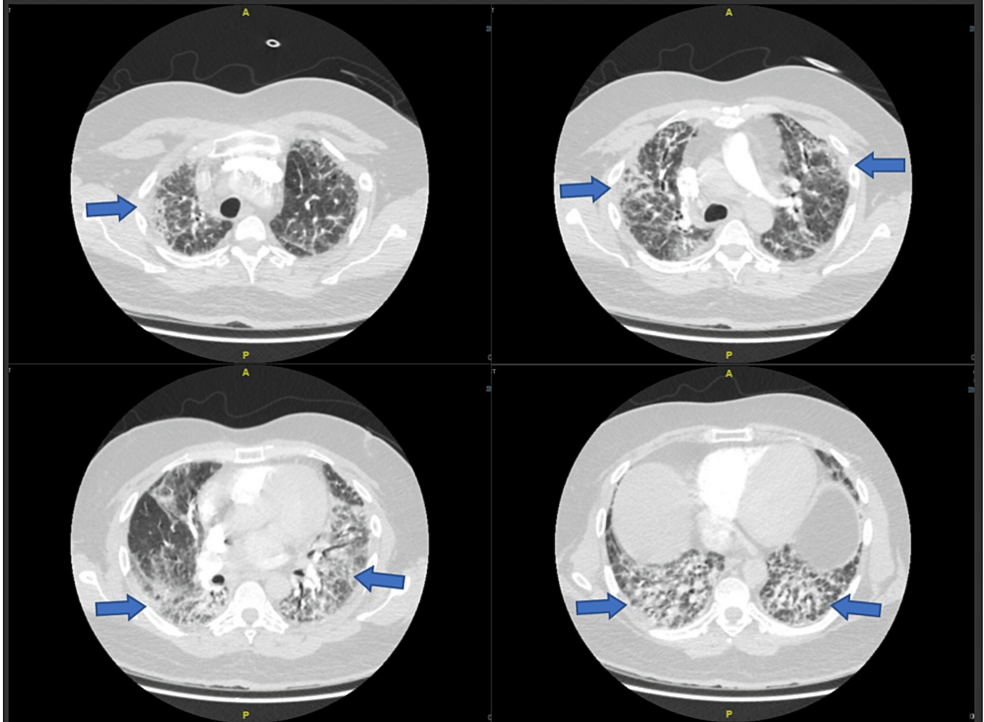
Patient’s chest computed tomography showing fibrotic nonspecific interstitial pneumonia pattern

Additionally, echocardiogram was unremarkable, and chest computed tomography (CT) was planned. Later on the same day of admission, the patient's condition deteriorated, and he required 5 liters/min of oxygen to maintain a saturation of 91%. Urgent CT angiography was done, which ruled out pulmonary embolism; nonetheless, it showed lung parenchymal changes most likely representing fibrotic nonspecific interstitial pneumonia (NSIP) (Figure 3).

**Figure 4:**
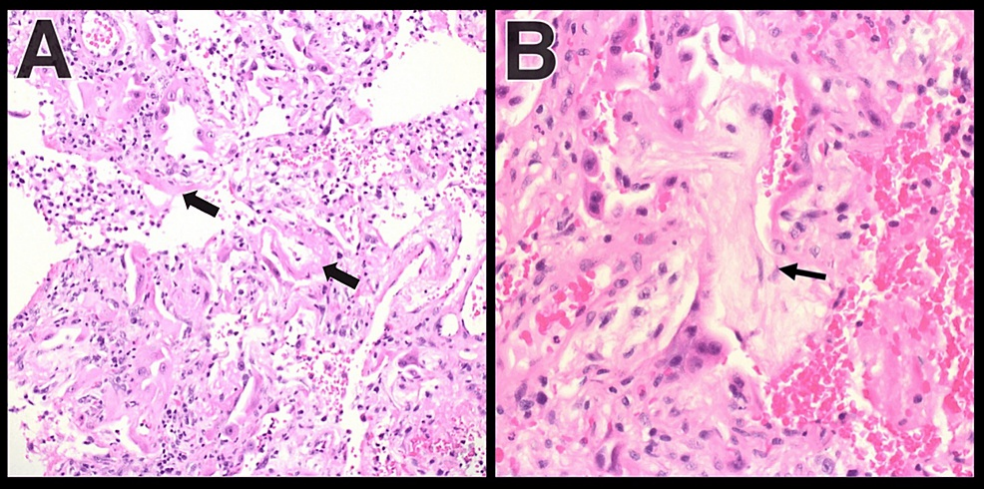
(A) Hyaline membrane and reactive pneumocytes with mixed inflammation and (B) organizing features and adjacent reactive pneumocytes

Due to rapidly worsening respiratory failure, the patient required intubation and mechanical ventilatory support. The clinical features and the speed of the patient's deterioration raised the concern of clinically amyopathic dermatomyositis (CADM), so anti-melanoma differentiation-associated gene 5 (anti-MDA5) and anti-Ro52 were sent. When the results were pending, the patient's condition worsened on the maximum ventilation settings, thus mandating to start the patient on extracorporeal membrane oxygenation (ECMO). A follow-up CXR after ECMO cannulation revealed a large left pneumothorax and pleural effusion. Afterward, the patient underwent chest tube insertion and bedside open lung biopsy (BOLB). The results of anti-MDA5 and anti-Ro52 came back positive, and the biopsy showed proliferative features with diffuse alveolar damage (Figure 4).

**Figure 5:**
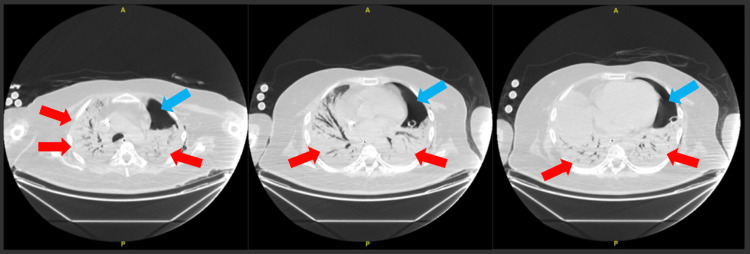
Repeated chest imaging showing new bilateral extensive airspace changes (red arrows) with large left pneumothorax (blue arrows)

The findings confirmed CADM's diagnosis with RP-ILD, and he was started on cyclophosphamide 1 g single dose. A week later, the patient's condition did not improve, and he started to have pancytopenia with WBCs of 0.5 x 10^9^/L, neutrophils count of 0.42 x 10^9^/L, hemoglobin (Hgb) of 10.9 x 10^9^/L, and platelet of 34 x 10^9^/L. The patient was started on G-CSF 300 mcg for seven days, which resulted in count recovery. The patient continued to remain on ECMO support without any improvement, and repeated chest CT showed new bilateral extensive airspace changes, suggestive of acute respiratory distress syndrome (ARDS) with large left pneumothorax (Figure 5).

The patient's condition kept declining as he developed pneumonia with parapneumonic effusion, candidemia, and acute kidney injury requiring continuous venovenous hemodialysis (CVVHD). The patient reached maximum vasopressor support and ECMO support, but two days later, the patient passed away.

## Discussion

This case presented a young gentleman with two years of intermittent itching that became constant in the last four months and later developed night sweating and subjective fever. The patient’s condition rapidly deteriorated as he developed ILD requiring ECMO. Lung biopsy and blood work attributed the symptoms to CADM.

CADM is a systemic inflammatory condition in which patients suffer from cutaneous features that may last for two years before unveiling myositis [13]. In our case, the patient's rash developed a few months before his presentation; however, he was having itching for two years, which may be a sign of CADM. Moreover, the patient did not complain of any muscle weakness, and his initial CK was normal, which is the case in most CADM patients as most cases have normal muscle enzyme levels and even normal muscle biopsy, although a slight elevation in CK is possible [13]. An essential point in the patient's history is that he complained of some constitutional symptoms raising suspicion of malignancy. Although those constitutional symptoms may occur in various conditions, including autoimmune disorders, multiple studies suggested an association between CADM and malignancies [14-16]. Other than our patient's symptoms, his labs and imaging did not reveal malignant disease. The main findings were RP-ILD related to CADM.

The prevalence of ILD is more remarkable in CADM patients than in patients with classic dermatomyositis or polymyositis [17]. Around 13% of patients with CADM develop ILD, while a smaller portion will suffer from RP-ILD [7]. According to the European Respiratory Society and American Thoracic Society, RP-ILD is a progressive deterioration associated with ILD within three months [18]. RP-ILD is the leading cause of mortality in CADM patients alongside pneumomediastinum [19]. The most evident radiographic finding is a diffuse reticulonodular pattern with patchy bilateral ground-glass opacities [19]. The pathological hallmark in patients with RP-ILD with positive anti-MDA5 antibodies is the diffuse alveolar damage seen on lung biopsies [20]. It is proposed that the lung damage in CADM patients may be due to anti-MDA5 antibodies directly rather than a secondary effect alone [21].

In the presented case, both anti-MDA5 and anti-Ro52 antibodies were detected. These two antibodies’ dual presence significantly lowers the patients' survival rate compared to single anti-MDA5-positive patients [22]. Since our patient was fragile and ECMO-dependent, a BOLB was performed to reach a diagnosis while the test results returned. BOLB is indicated in cases where pulmonary fibrosis is suspected [23]. Nonetheless, ECMO requires systemic anticoagulation to prevent clotting, but tragical adverse events may occur under these circumstances. The most worrisome complications of such a strategy are air leaks and hemothorax. Data about BOLB in adult patients on ECMO are limited. Rozé et al. reported five cases where none of them had air leaks, and two suffered from bleeding requiring transfusion [24]. In our case, the patient had a CT after the biopsy showing a large pneumothorax with no signs of bleeding, but this finding was present before the BOLB.

There is no consensus regarding the treatment of CADM. Multiple studies have reported various medications, including steroids, immunosuppressants, or immunomodulators such as cyclophosphamide, cyclosporine, methotrexate, mycophenolate, and tacrolimus [19,25,26]. However, the prognosis is poor, and the mortality remains high. Nonetheless, plasma exchange and polymyxin B hemoperfusion showed promising results, especially that polymyxin B hemoperfusion may result in anti-MDA5 antibody titer reduction [27-29]. Recently, Romero-Bueno et al. published a study that recommended a combination therapy to treat RP-ILD in patients with positive anti-MDA5 antibodies [30]. The recommended combination included glucocorticoids plus a calcineurin antagonist with or without intravenous cyclophosphamide. Immunosuppressive medication such as cyclophosphamide and/or mycophenolate mofetil can be used in addition to rituximab in cases when calcineurin antagonists cannot be used. On the other hand, the authors did not recommend using azathioprine, methotrexate, and leflunomide as induction therapy in patients with RP-ILD and positive anti-MDA5 antibodies [30].

## Conclusions

We presented a case of a gentleman who was diagnosed with RP-ILD secondary to CADM. The patient had rapid deterioration of his condition and was treated with cyclosporine. The patient's condition did not improve and he passed away.

CADM with RP-ILD is a fatal disease that requires early detection and intervention. The diagnosis can be challenging and mandates invasive intervention. The presence of anti-MDA5 and anti-Ro52 antibodies simultaneously with evidence of alveolar hemorrhage in a lung biopsy indicates a poor outcome. Nonetheless, detection of these antibodies is considered diagnostic for CADM with RP-ILD. Also, BOLB may be considered a reasonable option in patients who are on ECMO. Furthermore, studies would be beneficial to establish the safety of this approach.
